# Counterirritation by Pain Inhibits Responses to and Perception of Aversive Loud Tones

**DOI:** 10.1177/00315125231183604

**Published:** 2023-06-20

**Authors:** Silvia Metzger, Claudia Horn-Hofmann, Stefan Lautenbacher

**Affiliations:** 1 Department of Physiological Psychology, 240375 Otto-Friedrich-University of Bamberg, Bamberg, Germany

**Keywords:** counterirritation, pain inhibition, pain specificity, aversiveness, unpleasantness

## Abstract

The application of a noxious stimulus reduces the perception of other noxious stimuli, which can be assessed by an experimental method called “counterirritation.” The question arises whether this type of inhibition also affects the processing of other aversive (but not nociceptive) stimuli, such as loud tones. If aversiveness or, in other words, negative emotional valence qualifies a stimulus to be affected by counterirritation, the general emotional context may also play a role in modulating counterirritation effects. We involved 63 participants in this study (*M* age = 38.8, *SD* = 10.5 years; 33 males, 30 females). We tried to counterirritate their perceptual and startle reactions to aversively loud tones (105 db) by immersing the hand into a painful hot water bath (46°C) in two emotional valence conditions (i.e., a neutral and a negative valence block in which we showed either neutral pictures or pictures of burn wounds). We assessed Inhibition by loudness ratings and startle reflex amplitudes. Counterirritation significantly reduced both loudness ratings and startle reflex amplitudes. The emotional context manipulation did not affect this clear inhibitory effect, showing that counterirritation by a noxious stimulus affects aversive sensations not induced by nociceptive stimuli. Thus, the assumption that “pain inhibits pain” should be widened to “pain inhibits the processing of aversive stimuli.” This broadened understanding of counterirritation leads to a questioning of the postulate of clear pain specificity in paradigms like “conditioned pain modulation” (CPM) or “diffuse noxious inhibitory controls” (DNIC).

## Introduction

The investigation of pain inhibition has gained a recent boost in interest because (a) pain inhibition has seemed to qualify as a major risk factor for clinically significant chronic pain, and (b) valid, reliable, and inexpensive psychophysical assessment methods have become available (Fernandes, et al., 2019; Kennedy, et al., 2016; O’Brien, et al., 2018; Ramaswamy & Wodehouse, 2021). These approaches to assessment have been summarized as a tool to assess “conditioned pain modulation (CPM)” (Nir & Yarnitsky, 2015). The CPM paradigm implies pain specificity because both the conditioning stimulus that activates inhibition and the test stimulus that indicates inhibition are assumed to be painful. However, avoiding this restricted assumption of two pain-related stimuli, we prefer the term “counterirritation,” which denominates a more broadly understood inhibitory action of one stimulus upon another and which has also a long tradition of use in this context. There have been experimental demonstrations that counterirritation is not necessarily pain-specific, as perceptions of subjectively non-painful stimuli have also been inhibited in the context of painful counterirritation ((Horn et al., 2012; Kunz et al., 2006; Lautenbacher et al., 2002; 2007; 2008; Pielsticker et al., 2005; Rustamov et al., 2016; Talbot et al., 1987). However, investigators have not yet widened the counterirritation concept so far as to show whether counterirritation affects aversive sensations in general (e.g., aversively loud tones), including sensations not produced by any noxious stimuli. To our knowledge, no investigators have systematically determined whether counterirritation can inhibit the perception or responsiveness to non-noxious, aversive stimuli. We found only one early study of pain effects on itching as a kind of predecessor of this research thread (Murray & Weaver, 1975). Since there is a neurophysiological overlap between pain and itch, we are not sure how to classify this study.

In the present study, we attempted to counterirritate participants with immersion of the hand in painful hot water and then measure inhibition of both responsiveness to and perception of aversive loud tones. If the essential feature of a stimulus to be affected by counterirritation is its negative emotional valence (i.e., aversiveness) and not painfulness produced by noxious events, the emotional context may matter during the stimulus test. Therefore, we studied counterirritation in two contexts, one with a neutral emotional valence and one with a negative emotional valence. To induce emotional valence, we followed the example of many prior researchers who had participants view emotionally valenced pictures (e.g., Domínguez-Borràs et al., 2008). We used the participants’ startle blink reflex to aversive tones and the participants’ ratings of the loudness of the aversive tones as indicators of aversiveness inhibition. The startle blink reflex is an automatic defensive reaction to sudden intense stimuli like aversive tones (Lang et al., 1990); and it is modulated by positively or negatively valenced stimuli. We assumed that counterirritation by pain would inhibit responsiveness to and perception of aversive tones, as indicated by a decrease in startle reflex amplitudes and reduced loudness ratings. Moreover, we hypothesized that the inhibitory effect of pain on the responsiveness to and perception of aversive tones would be abolished or dampened in a negatively valenced context, but not in a neutrally valenced context. We intended to show that counterirritation triggered by pain would affect other aversive but non-noxious stimuli (aversive tones) and that the emotional context of the stimulation would modulate its effect.

## Method

### Participants

We recruited participants by advertisements (wall posters, social media) at the University of Bamberg. Interested persons were asked to send us an email, signaling their readiness to undergo an informed consent process and further screening. All participants provided written informed consent, including an understanding of their right to withdraw from the study at any time, before engaging in any experimental procedures. All participants received either monetary compensation for their participation or course credits (psychology students). We obtained approval for the study protocol from the ethics committee of the University of Bamberg and can state that this ethic committee meets all conditions set in Germany by the Deutsche Forschungsgemeinschaft (DFG) to examine and approve studies including aversive stimulation.

Sixty-seven healthy, pain-free individuals between 20–54 years of age participated in this study (*M* age = 38.8, *SD* = 10.6 years; 34 males, 33 females). We based this sample size on a power analysis calculation (Sample Power 2.0, SPSS Inc., Chicago, IL, USA) in which we assumed a moderate effect size due to previous findings on CPM effects (Horn et al., 2012; Lautenbacher et al., 2008). Other assumptions in this power analysis were 80% power, and statistical significance of *p* < .05. This calculation yielded an estimated required sample size of 60 participants. We recruited 67 participants to allow for possible attrition and missing data.

Prior to the experiment, which was carried out in a laboratory of the University of Bamberg, we conducted a standardized phone interview with each participant to exclude those with acute and chronic mental or physical diseases. No participant was allowed to take any analgesic medication or alcohol at least 24 hours prior to the test sessions. On the experimental day, participants were requested to refrain from drinking coffee. Smokers were allowed to continue normal smoking habits to avoid nicotine withdrawal, but they were requested to stop smoking one hour before the start of the experiment to prevent nicotinic influences on arousal with consequences on our results.

### Research Design

In this study, we sought to answer whether a painful counterirritant (hot water) would affect processing of aversive stimuli (loud tones) and whether the emotional context (emotional pictures) of the test procedure would modulate this effect. We used a 2 × 2 within-subjects design, in which we analyzed both the factors “counterirritation” (baseline vs. hot water) and “emotional context” (neutral vs. negative).

### General Procedures

Prior to the experiment, after participants gave their informed consent, they were administered the “Mini Dips,” a short structured diagnostic interview for mental disorders, to exclude participants with mental diseases (Margraf et al., 2017). Thereafter, participants were prepared for the startle reflex measurement by attaching three electrodes around the participant’s left eye.

During the experimental session, participants were seated in a comfortable chair in front of a computer display. Our experimental session included two independent variables, namely “counterirritation” and “emotional context.” For assessing *counterirritation* effects, aversive tones were first applied in a baseline condition and thereafter in the counterirritation condition. The counterirritant was immersion of the right hand in hot water. The sequence of baseline followed by counterirritation (together one block) was repeated once (resulting in two blocks). The two blocks differed by the emotional context presented in parallel. *Emotional context* was manipulated by presenting either aversive pictures showing burn wounds (negatively valenced block) or neutral pictures showing everyday objects (neutrally valenced block). Thus, there were four conditions arranged in two blocks: (a) baseline with an emotionally valenced context (neutral or negative) followed by counterirritation with a paralleled emotionally valenced context (neutral or negative); and (b) baseline with an emotionally valenced context (negative or neutral) again followed by counterirritation with a paralleled emotionally valenced context (negative or neutral). The changing order of the two emotionally valenced blocks (i.e., neutral and then negative or negative and then neutral) was randomized but across participants in a balanced distribution. There were 10-minute breaks between conditions. Primary outcome measures (dependent variables) were participants’ ratings of the loudness of aversive auditory tones and the amplitude of their startle reflex to the aversive tones; a secondary outcome measure was the threat rating of the emotional pictures as a manipulation check. Participants were informed about the experimental stimulation (hot water, loud tones and emotional pictures) but not about their exact timing.

To familiarize participants with the stimuli (aversive and neutral pictures, aversive tones, hot water bath) and the rating procedure, we administered practice trials before the actual measurement started. At this time point, the participants already knew that all stimuli were established experimental tools in many laboratories worldwide, and that they had been used only after ethical approval from the oversight committees in past studies. Overall, the experiment (without preparation and debriefing) lasted about 70 minutes. After the experiment, participants were given a full explanation of the study aims and were specifically asked for information about their discomfort, any other problems they encountered, and their suggestions for the future.

### Measures

#### Implementation of Counterirritation and Emotionally Valenced Context

**Figure 1.**
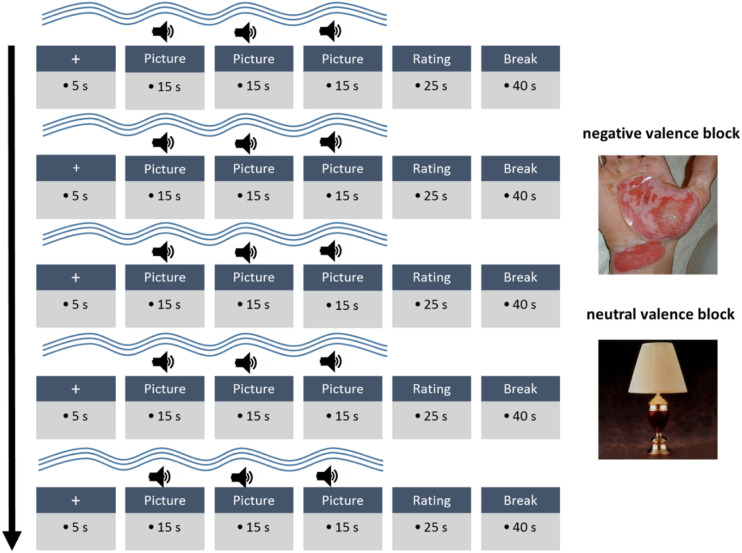
Implementations of Counterirritation and an Emotional Valence Context. *Note*: Only a condition with counterirritation is shown; the hot water immersion is indicated by the wavey line.

Each condition (baseline_neutral context_, counterirritation_neutral context_, baseline_negative context_, counterirritation_negative context_) consisted of five sets of 75 second trials, each separated by a 40 second break (meaning that each condition took nine minutes). Each trial started with a fixation cross displayed on a computer screen for five seconds. Next, there was a 45 second pain stimulation in which the aversive tones and pictures were presented. The trials were always completed with an additional 25 second period in which participants rated the tones (see Figure 1). In the two counterirritation conditions (counterirritation_neutral context_, counterirritation_negative context_), an instruction was displayed below the fixation cross: ‘please immerse hand now.’ Within each immersion interval, three pictures were presented for 15 seconds each (resulting in 15 pictures in each condition, or 60 pictures overall). Three tone presentations occurred within an interval from 8–13 seconds after each picture onset. After each stimulation interval, rating scales were presented on the screen (see Figure 1). Loudness ratings and threat ratings were assessed after each stimulation interval. Moreover, the painfulness of the hot water bath was assessed at the same time by participant ratings, but only in the conditions with counterirritation. In Figure 1, the conditions with counterirritation are graphically depicted.

#### Stimuli

##### Aversive Tones

Auditory stimuli with aversive loudness were short noise bursts (105 db, 50 ms) presented binaurally over headphones above constant background noise applied for masking (68 db). These tones are known to be clearly aversive and suitable to trigger the startle reflex (Horn et al., 2012). However, the guidelines published for startle reflex studies by Blumenthal et al. (2005) highlighted that these stimuli are safe. In each of the four conditions (baseline_neutral context_, counterirritation_neutral context_, baseline_negative context_, counterirritation_hnegative context_) 15 aversive tones were presented, resulting overall in 60 aversive tones.

##### Hot Water Counterirritant

We used a water bath apparatus (Variostat, Huber; Germany) for counterirritation with painful hot water at 46°C (Horn-Hofmann et al., 2016). To avoid a regional temperature difference within the water bath and temperature layers of different intensities around the immersed hand, the water was stirred with a force and suction pump. Only during the counterirritation conditions were participants instructed via the computer screen to immerse their right hand into the hot water during the presentation of the fixation cross and stimulation interval and to remove the hand when rating scales appeared until the next trial started. In total, the participants immersed their hands for 225 seconds into hot water but never continuously; the five immersion phases lasted 45 seconds each, with no-stimulation breaks of 65 seconds in-between. The stimulation time of 45 seconds is shorter than the usual ones, which have already been shown to be tolerable and harmless (Horn-Hofmann et al., 2016; Lautenbacher et al., 2008). After each immersion phase, participants rated the painfulness of the hot water bath (“0 = not at all painful” to “10 = very painful”) (see Figure 1). Pain ratings were averaged across the five ratings in the two conditions (counterirritation_neutral context_, counterirritation_negative context_).

##### Pictures for Emotional Context

For creating a context with negative valence, we used negative photographs compared to neutral pictures. The negative context pictures were intended to augment counterirritation by painful hot water due to a content-related association. The negative pictures showing burn wounds were pre-selected from a pool of pictures with neutral and negative valence. The neutral pictures were taken from the International Affective Picture System (IAPS; Lang at al., 2005), according to the following three criteria, (a) neutral content (e.g., household objects, vehicles; no people); (b) valence ratings of ≥ 4 and ≤6; and (c) arousal ratings ≤ 4. Aversive pictures were taken from the internet, based on picture content. We used only pictures showing burn wounds of the hand, found in a Google image search with the keywords “burn“ or “burn wound“ in various languages (English, German, Dutch and French). All pictures were proportionally altered to be of the same size (about 13 × 9 cm). This pre-selection procedure resulted in 40 neutral and 60 aversive pictures.

In a pilot study with separate informed consent from all participants, 30 participants (psychology students; 19 female and 11 male) rated the valence and their arousal for each of the 100 pictures, using the Self-Assessment Manikin scale (SAM; Lang, 1980) on which ratings can range from 1 to 9, with 9 indicating most positive valence or highest arousal. The following criteria were applied for final selection: Neutral pictures were required to have mean valence ratings of 5 ± 1 and mean arousal ratings ≤ 2.5; aversive pictures were required to have mean valence ratings ≤2.5 and mean arousal ratings ≥ 5.5. Outliers from the respective picture category (neutral or aversive) were excluded. The final picture set consisted of 60 pictures. The 30 neutral and the 30 aversive pictures were each randomly split in two subsets of 15 pictures as emotionally valenced context for the four experimental conditions. The participants were informed about the content of the pictures (wounds, neutral scenes) and repeatedly informed that they could make use of their right of withdrawal at any time. In addition, participants with stressful phases in their present life and any mental disorders that would sensitize them to our procedure, as assessed by the DIPS, had already been excluded. After years of using emotional pictures with negative valence in experiments, others concluded that the affection reactions are transient and harmless (Bradley & Lang, 2007).

To assess whether the burn pictures were perceived as threatening in the present study, participants were asked to rate the perceived threat of the pictures (“0 = not threatening” to “10 = extremely threatening”) after each stimulation interval (5 times per condition) (see Figure 1). Threat ratings were averaged across the five rating assessments in each of the four conditions (baseline_neutral context_, counterirritation_neutral context_, baseline_negative_ _context_, counterirritation_negative context_). Successful emotional context manipulation was evidenced by threat ratings that were higher in the “negative context block” compared to the “neutral context block.

#### Dependent Variables - Response Inhibition Measures

##### Startle Reflex

We measured the participants’ startle blink reflex by recording surface EMG activity from the musculus orbicularis oculi beneath the left eye (recording device: SIGMA Plpro/Type Databox DB 36; SIGMA Medizin-Technik GmbH, Gelenau, Germany, including a 16 bit AD convertor with a dynamic range from 0.5 μV to 2 mV). After cleaning the skin, we placed two 4 mm recording electrodes (Ag/AgCl) on the skin surface overlaying the orbicularis oculi muscle and attached a ground electrode at the forehead. The recording bandwidth of the EMG signal was between 0.2 Hz and 300 Hz. The input resistance was above 20 mΩ and the signal was sampled at a rate of 512 Hz. Triggers were automatically set to mark the onset of the aversive tones to allow the event-related analysis of signals. After recording, data were analyzed offline with “Vision Analyzer” (Brain Products, Munich, Germany). First, the signal was divided in segments, each containing one EMG response to one aversive tone. Analysis included filtering of the signal (50 Hz notch filter, 20 Hz high-pass filter and 256 Hz low-pass filter) as well as rectifying and integrating the signal. Integration was executed over a time interval from 0 to 250 ms after tone onset. Responses without peaks between 30 and 100 ms after tone onset were excluded just like trials with responses that did not fit the typical shape of a startle response. Overall 4.4% of responses were excluded (percentage of excluded responses did not differ between blocks or conditions). Missing values were substituted by means, which were calculated from the available data for each individual. Startle reflex amplitudes were defined as voltage differences between the average baseline and voltage peak within a time frame of 30–100 ms after tone onset. Our measures and analyses were based on recommendations by Blumenthal et al. (2005) and were successfully applied in several previous studies (Horn et al., 2012; Horn-Hofmann & Lautenbacher, 2015; Horn-Hofmann et al., 2016). Startle reflex amplitudes were averaged across the 15 startle reflex assessments in each of the four conditions separately (baseline_neutral context_, counterirritation_neutral context_, baseline_negative context_, counterirritation_negative context_). To investigate if the painful counterirritant suppressed the responsiveness to the aversive tones, we compared the startle reflex amplitudes between the conditions at baseline and counterirritation. A decrease in startle reflex amplitudes during counterirritation indicated inhibition.

##### Loudness Ratings of the Aversive Tones

After each stimulation interval (five times per condition) participants rated the perceived loudness of the aversive tones on a numerical rating scale ranging from “0 = no noise” to “10 = very loud noise” (see Figure 1). Loudness ratings were averaged across the five rating assessments in each of the four conditions (baseline_neutral context_, counterirritation_neutral context,_ baseline_negative context_, counterirritation_negative context_). To investigate whether the painful counterirritant inhibited the perception of the aversive tones, we compared loudness ratings between baseline and counterirritation. A decrease in loudness ratings during counterirritation indicated inhibition.

### Statistical Analyses

To test our hypotheses, we calculated two separate 2 (Condition: baseline, counterirritation) × 2 (Block: neutral context, negative threat) repeated measures analyses of variance (ANOVAs), one with loudness ratings and one with startle reflex amplitudes as dependent variables. Inhibitory effects would be proven by a main effect of Condition and a modulating effect of emotional valence context by an interaction between Condition and Block. As a manipulation check of whether the pictures were apt to induce a neutral or negative emotional context, we analyzed threat ratings by a repeated measurement ANOVA with ‘Block’ (neutral context, negative context) and ‘Condition’ (baseline, counterirritation) as within-subject factors. If the ANOVA revealed a significant interaction effect, tests of simple main effect were conducted as post-hoc tests (paired t-tests). When the assumption of sphericity was violated, a Greenhouse-Geisser correction was used. To test if the painfulness of the hot water bath unwantedly differed between the two blocks (neutral context and negative context), we calculated a paired *t* test. All data were analyzed using the Statistical Package for the Social Sciences (SPSS, v. 28; IBM Corp., Armonk, NY). Statistical significance was set at α = 5%.

## Results

### Participants

Of the 67 participants in our study, we excluded one for technical problems and three who were unable to tolerate the hot water bath (*n* = 3). The demographic characteristics for the remaining 63 participants included in our analyses are displayed in Table 1.

**Table 1.** Participant Characteristics.

*N*	63
Age in years; mean (*SD*)	38.8 (10.5)
Gender (*N* ♂/♀)	33/30
BMI: mean (*SD*)	23.7 (3.5)
Education	
Lower secondary degree (*N*)	1
Middle secondary degree (*N*)	12
Higher secondary degree (*N*)	25
University degree	25
Relationship status (single/married)	35/22

### Manipulation Check

#### Emotional Context

**Figure 2.**
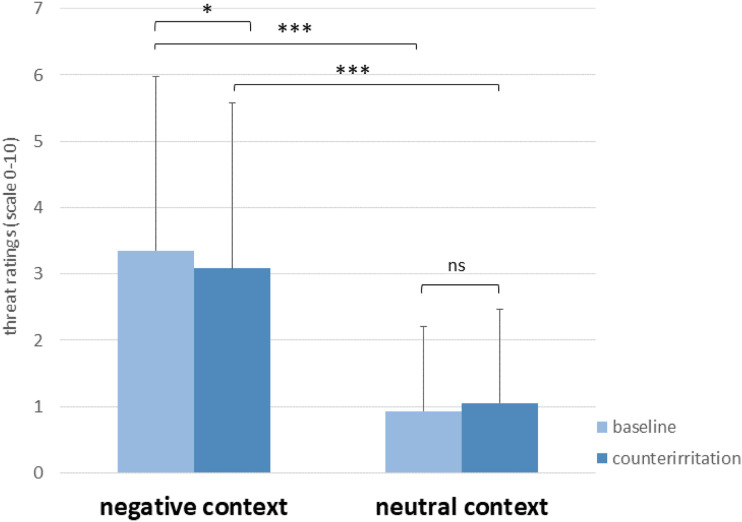
Participants’ Mean Threat Ratings in the Negative Emotional Context Block and the Neutral Emotional Context Block During Baseline and Counterirritation Conditions. *Note:* ***p < .001, *p < .05, ns = not significant; error bars represent standard deviations.

We first evaluated whether the manipulation of emotional context by showing neutrally and negatively valenced pictures, intended to be perceived as threatening, was successful. Participants’ threat ratings are shown in Figure 2. The ANOVA yielded a significant main effect of Block (see Table 2), with higher threat ratings in the negative context block compared to the neutral context block. There was no significant main effect of Condition (baseline vs. counterirritation) but there was a significant interaction between Condition x Block. Post-hoc tests (*t* test) revealed that threat ratings were significantly higher at baseline than during counterirritation only in the negative context block (*p* = .017) but not in the neutral block (*p* = .180). Most important for the manipulation check, post-hoc tests (*t* test) also revealed that threat ratings were higher in the negative context block compared to the neutral block for both baseline (*p* < .001) and counterirritation (*p* < .001) conditions, indicating that the emotional context manipulation was successful.

**Table 2.** Participant ANOVA Results for Manipulation Check (Threat Ratings) and Main Outcomes of Startle Reflex (Amplitudes) and Aversiveness Ratings (Tones Loudness).

Factor	DF	Sum of Square (SS)	Mean Square (MS)	F	*η* ^*2*^
Manipulation check - emotional context: Threat ratings					
‘Condition’ *(baseline* vs. *counterirritation)*	1	0.32	0.32	0.79	*0.012*
‘Block’ *(negative threat* vs. *neutral context)*	1	312.45	312.45	53.41^c^	*0.464*
‘Condition’ x ‘block’	1	2.48	2.48	9.92^b^	*0.143*
Error	62	15.49	0.25		
Main outcome: Startle reflex amplitudes					
‘Condition’ *(baseline* vs. *counterirritation)*	1	931.83	931.83	13.12	*0.175*
‘Block’ *(negative threat* vs. *neutral context)*	1	19.03	19.03	0.26	*0.004*
‘Condition’ x ‘block’	1	23.98	23.98	0.48	*0.008*
Error	62	3108.00	50.13		
Main outcome: Loudness ratings of aversive tones					
‘Condition’ *(baseline* vs. *counterirritation)*	1	9.53	9.53	15.54^c^	*0.200*
‘Block’ *(negative threat* vs. *neutral context)*	1	1.09	1.09	1.80	*0.028*
‘Condition’ x ‘block’	1	0.01	0.01	0.02	*0.001*
Error	62	22.74	0.37		

*Note:*

^a^indicates significance at α = .05.

^b^indicates significance at α = .01.

^c^indicates significance at α = .001

#### Pain Ratings for Hot Water Hand Immersion (Counterirritant)

The hot water bath counterirritation was perceived as moderately painful in both conditions (counterirritation_neutral context_ and counterirritation_negative context_). There were no significant differences in pain ratings between the negative context block (*M* = 3.62, *SD* = 2.18) and the neutral context block (*M* = 3.53, *SD* = 2.14).

### Response Inhibition Measures

#### Startle Blink Reflex Amplitude

**Figure 3.**
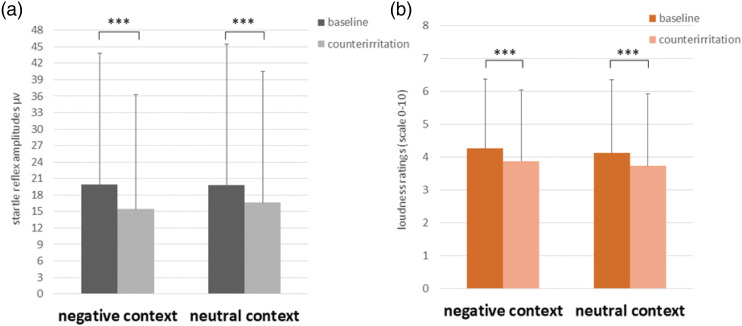
Participants’ Mean Startle Blink Reflex Amplitudes (a) and Loudness Ratings (b) in the Negative Emotional Context Block and the Neutral Emotional Context Block During Baseline and Counterirritation Conditions. *Note:* ***p < .001, error bars represent standard deviations.

Startle blink reflex amplitudes are shown in Figure 3(a). The ANOVA showed a main effect for Condition (baseline vs. counterirritation) on startle reflex amplitudes (see Table 2). As can be seen in Figure 3(a), startle reflex amplitudes were significantly decreased during counterirritation compared to baseline. However, there was no main effect of Block and no significant interaction between Condition x Block, suggesting no effects of emotional context.

#### Ratings of Aversive Tones

Loudness ratings of aversive tones are displayed in Figure 3(b). The ANOVA showed a main effect of Condition (baseline vs. counterirritation) on loudness ratings (see Table 2). As can be seen in Figure 3(b), loudness ratings were significantly lower during counterirritation compared to baseline. There was no main effect of Block and no significant interaction effect, suggesting no effects of emotional context.

## Discussion

We investigated whether counterirritation by pain inhibited responsiveness to and perception of non-noxious aversive stimuli. Our results showed that painful hot water reduced both our participants’ startle blink reflex amplitudes to and their loudness ratings of aversive but non-nociceptive tones. This finding loosens specificity assumptions associated with some formulations of the ‘conditioned pain modulation’ (CPM) theory or the ‘diffuse noxious inhibitory controls’ (DNIC) theory asserting that ‘pain inhibits pain,’ by showing that ‘pain also inhibits the processing of other aversive but non-nociceptive stimuli.’ If this finding can be replicated in future studies, counterirritation by pain would seem to act super-modally on all perceptions associated with an aversive valence. In other words, pain may act on brain centers and psychological mechanisms that detect the emotionality of perceived stimuli and thereby regulate the inhibition of sensations with negative valence. This assumption would not exclude previously established pain-specific inhibitory actions in counterirritation, but this broader conception of inhibition allows for wider perceptual interactions with sensations that carry negative valence not produced by noxious events.

Several mechanisms might explain the interaction between negatively valenced sensations beyond the pain-inhibitory action as described by DNIC (Le Bars et al., 1991). For example, the idea of a potentiation of defensive reactions according to ‘motivational priming theory’ postulates that responses to multiple stimuli that are emotionally congruent are enhanced (Lang et al., 1998). This theoretical assumption would advocate for facilitation but not inhibition. Algom et al. (1986; 1987) demonstrated, in contrast to our findings, that ‘painful’ electrical current and loud tones concurrently applied produced a summation of stimulus effects. Prior DNIC and CPM research showed that inhibition mainly occurred when the inhibiting stimulus of the two applied stimuli was intense, tonic and precedent, and these factors were not present in the studies by Algom and colleagues, who used only phasic stimuli in parallel. Algom and colleagues argued for a functional theory of pain, based on their data; however, this theory helps – as suggested above - to explain facilitatory but not inhibitory effects. Pain adaptation-level theory seems to better fit our data; this theory observes that the evaluation of a stimulus depends on the individual’s history of stimulation (Helson, 1947; Rollman, 1979). For example, the intense painfulness of a strong stimulus can serve as a point of reference and may result in a later stimulus being perceived as weak; whereas the opposite may happen if the point of reference was set by a weak stimulus (Lautenbacher et al., 2007). In our study, the hot water immersion, which participants rated as moderately painful, may have served as such a strong stimulus reference point, leading to a reduced perception of negative valence for the relatively less intense aversive tones. The most recent contributions to explaining pain facilitation and inhibition due to the interaction of several stimuli have been derived from theoretical accounts of multisensory integration. As demonstrated, the magnitude of behavioral facilitation and modulation of neural activity is inversely related to the strength of the presented stimuli. This would mean that interacting weak stimuli lead to strong facilitation whereas the interaction of strong stimuli does the opposite and may take the form of inhibition. The principle describing this effect is called ‘inverse effectiveness’ (IE) (Pomper et al., 2013; Rach et al., 2011 Senkowski et al., 2014). Since both stimuli applied in the present study were intense, a mutual weakening of stimulus effects up to inhibitory effects – as we observed - is likely.

We also investigated the influence of emotional context on the inhibitory effect of pain on subsequent stimuli. Although our manipulation check showed that we successfully presented negatively valenced threatening pictures (burn wounds) that differed from our neutrally valenced non-threatening pictures (everyday objects), neither the main effect of emotional context nor its interaction with counterirritation was significant. Thus, we could not determine any effect of the pictures with a negative valence on the participants’ responsiveness (startle blink reflex) to and perception (loudness rating) of loud tones. Such a complete failure of modulation by positively and negatively valenced pictures is unusual in pain studies (Kenntner-Mabiala & Pauli, 2005; Rhudy et al., 2008). While there have rarely been systematic studies of such emotional effects on the perception of tone loudness, hearing is strongly influenced by limbic brain structures (Jastreboff P. J. & Jastreboff M. M., 2004). Since our participant ratings showed only a moderate perceived threat, our emotional valence manipulation may not have been strong enough to trigger effects. Alternatively, participants may have been overchallenged because they had to process aversive tones, pain and emotional pictures in parallel. Since pain is a very imperative stimulus and was efficiently delivered in our experiment, participants may have been distracted from the pictures. Lastly a kind of emotional saturation may have occurred, due to summing three negative stimuli, namely aversive tones, pain and negatively valenced pictures.

In sum, we demonstrated an inhibitory effect of pain (hot water) on the processing of aversive auditory tones both on the physiological level (through a reduced amplitude of the startle reflex) and on the psychological level (through a reduced perception of the loudness of the aversive tones). Thus, counterirritation effects may occur if negative and valence congruent stimuli interact even in the absence of painful stimuli. This widens the concept of counterirritation, leaving the assumption of necessary pain specificity out of the picture and allows the inclusion of other aversively felt perceptions.

### Limitations and Directions for Further Research

There are important limitations to this study. First, the application of very short aversive tones (50 ms duration) in our study may cause problems in externally validating our findings, because we did not learn whether counterirritation by pain would also affect longer more tonic aversive stimuli. Furthermore, the question may arise whether our aversive tones unwantedly triggered physical pain, which would have invalidated our whole approach to counterirritation effects on aversive events in general but not only on pain. However, this is unlikely because the auricular pain thresholds are clearly above the intensity level of our sound stimulation (50 ms, 105 dB), which allows for the occurrence of discomfort but not for the occurrence of pain (Newman, 1972). Also, we tested only the modulation of counterirritation effects by emotional context using pictures eliciting threat. Other, negatively valenced pictures intended to trigger sadness, anger and anxiety still await evaluation. Finally, our small sample might be a limitation. Future studies should replicate these findings with larger participant samples and address these gaps in our methods.

## Conclusion

We found that pain inhibited responsiveness to and perception of aversive loud tones in a counterirritation paradigm. Thus, counterirritation effects may not be pain-specific but wider, affecting also the responses to and perception of other aversive stimuli not related to noxious events. Future studies should implement both pain-specific and non-noxious counterirritation designs, assessing effects on aversive perception triggered by pain and by other emotions and sensations with negative valence (startle, anxiety, anger, disgust, itch) for comparison.
